# Gestational experience alters sex allocation in the subsequent generation

**DOI:** 10.1098/rsos.160210

**Published:** 2016-07-13

**Authors:** A. M. Edwards, E. Z. Cameron, J. C. Pereira, E. Wapstra, M. A. Ferguson-Smith, S. R. Horton, K. Thomasson

**Affiliations:** 1 School of Biological Sciences, University of Tasmania, Hobart, Tasmania, Australia; 2 Department of Veterinary Medicine, University of Cambridge, Cambridge, UK; 3 Cytocell Ltd., Cambridge Technopark, Newmarket Road, Cambridge, UK

**Keywords:** sex allocation, maternal, paternal, fluorescent *in situ* hybridization, sex ratio

## Abstract

Empirical tests of adaptive maternal sex allocation hypotheses have presented inconsistent results in mammals. The possibility that mothers are constrained in their ability to adjust sex ratios could explain some of the remaining variation. Maternal effects, the influence of the maternal phenotype or genotype on her developing offspring, may constrain sex allocation through physiological changes in response to the gestational environment. We tested if maternal effects constrain future parental sex allocation through a lowered gestational stress environment in laboratory mice. Females that experienced lowered stress as embryos *in utero* gave birth to female-biased litters as adults, with no change to litter size. Changes in offspring sex ratio was linked to peri-conceptual glucose, as those females that had increasing blood glucose peri-conceptionally gave birth to litters with a higher male to female sex ratio. There was, however, no effect of the lowered prenatal stress for developing male embryos and their sperm sex ratio when adult. We discuss the implications of maternal effects and maternal stress environment on the lifelong physiology of the offspring, particularly as a constraint on later maternal sex allocation.

## 1. Introduction

Adaptive sex allocation hypotheses predict variation in the sex ratio of offspring where sex-specific fitness returns vary with local conditions and/or parental ability to invest [1–4]. Such hypotheses are logically appealing and have resulted in numerous empirical tests, including in mammals (reviewed in [5–7]). Initial reviews in mammals suggested little consistency in support for adaptive hypotheses, but methodological inconsistencies between studies explain some of the variation [5,7]. Nonetheless, unexplained variability both between and within species in empirical studies occurs, especially in mammals [8]. The unpredictability of effect sizes suggests that parents may be physiologically constrained in their ability to skew the sex of their offspring [9,10].

An increasing understanding of the underlying physiological mechanisms for maternal sex allocation suggests factors that might constrain maternal ability to skew sex ratios [10]. Lifelong and inter-generational modifiers of maternal physiology may constrain an individual's ability to respond to the current local conditions [10–12], particularly through maternal effects, the causal influence of the maternal phenotype or genotype on developing offspring [13–15]. Several factors have been linked to sex ratio skews through their physiological actions, including circulating glucose [5], testosterone [16–18] and stress hormones [19]. Each of these factors is influenced by the local conditions a mother experiences and can directly affect the developing fetus. Thus, the environment experienced *in utero* can alter physiological pathways, thereby changing the individual's response to the environment as adults [20]. Such maternal effects may result in parents that are physiologically constrained in their ability to alter sex ratios in response to current conditions.

Stress responses provide a link between the proposed mechanisms of sex ratio adjustment [19,21] and can have profound physiological impacts on developing offspring as a maternal effect [22]. Stressors experienced by the mother are mediated through internal hormone fluctuations; stressors stimulate the release of corticotropin-releasing hormone from the hypothalamus, which in turn stimulates the release of adrenocorticotropic hormone from the pituitary gland, resulting in the release of glucocorticoids (GCs; [23]). GCs then bind to receptors, which allow the body to return to homeostasis through acute stress events [23–25]. Fetuses are extremely sensitive to GCs [26,27], and so protective enzymes (e.g. 11 beta-hydroxysteroid dehydrogenase type 2) in the placenta metabolize roughly 80% of naturally occurring GCs, thereby buffering the fetus from high levels of GCs [28,29]. However, the remaining proportion can cross the placenta, and thereby influence offspring development [30]. These changes can be either deleterious or advantageous to the offspring (e.g. [31,32]) and can last a lifetime [31], potentially even persisting across generations [33,34]. Offspring fitness may be increased, for example by matching poor-quality mothers with reduced offspring demand [35] and offspring traits that increase survival [32]. However, changes that create a mismatch with the local environment are likely to result in offspring relatively less suited for the current environment, thus decreasing their fitness [36,37].

The physiological effects of maternal gestational stress on developing offspring include changes in the hypothalamic-pituitary-adrenal (HPA) axis function, immunity, glucose and insulin tolerance and regulation, body condition and adult reproductive behaviour and function in the offspring [38–40]. Stress probably influences maternal sex allocation, through increased susceptibility of male offspring to adverse conditions during late gestation [41], and more subtly through physiological changes persisting into adulthood. Changes to the HPA axis (and thereby sensitivity to stress) as a result of maternal effects during late gestation could influence offspring sex ratios and survival once that offspring itself reaches breeding age. Furthermore, such changes may influence maternal sex allocation through interactions with free glucose [5], because hepatic gluconeogenesis results from increased cortisol [42], and gestational stress can alter glucose levels and insulin tolerance lifelong [43,44]. Increases in peri-conceptual glucose increase the proportion of male offspring [5,45], due to interactions between free glucose and X-linked proteins and metabolic pathways [46], where female conceptus development is compromised under high glucose conditions [45,47] but enhanced under low glucose conditions. GCs also inhibit the secretion of reproductive hormones, including testosterone, also linked to sex ratio skews in mammals [48]. High levels of maternal testosterone have been linked to an increasing proportion of male offspring [49,50], hypothetically altering the receptivity of the egg to either X- or Y-chromosome-bearing spermatozoa in relation to follicular testosterone [17]. Hormonal differences between adult males have also been linked to variation in the X to Y ratio in sperm (reviewed in [9]) potentially also influencing paternal sex allocation. Therefore, maternal stress levels can influence offspring development during gestation in ways that could alter sex allocation when they reproduce, irrespective of current local conditions.

Here, we test if downregulated stress during late gestation in laboratory mice impacts (i) the physical development and reproductive success of offspring and (ii) their sex allocation, in terms of sperm sex ratios in adult males and birth sex ratios in females. We predict that offspring born to treated mothers will have an increased number of glucocorticoid receptors [51], and therefore increased susceptibility to stress [26]. Female offspring may then experience increases in offspring sex ratios as a result of increased gluconeogenesis [5]; however, we do not predict that these changes should influence male sperm sex ratios.

## 2. Material and methods

We used BALB/c mice bred and housed at the University of Tasmania, Australia. They were kept under 12 L : 12 D photoperiod in a temperature and humidity controlled room and provided with mouse chow (Barastoc® irradiated food) and filtered water ad libitum.

### 2.1. Generating focal females and males

**Figure 1.**
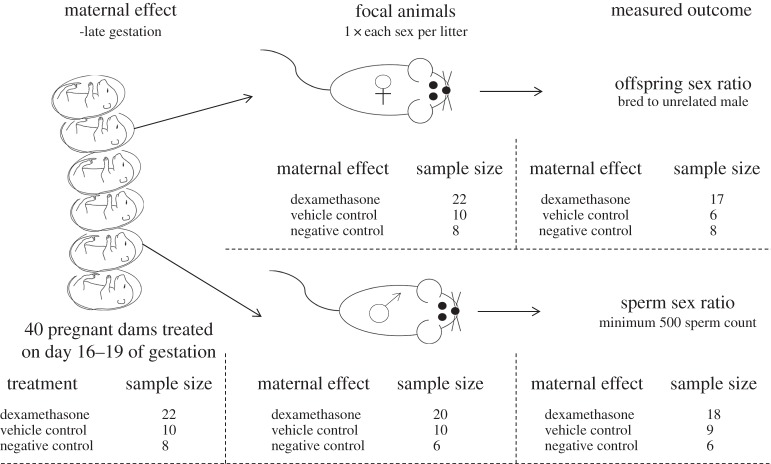
Diagram of the experimental design. The sample sizes at each stage of the experiment are listed based on treatment.

The experimental design is outlined in figure 1. Forty nulliparous dams were housed in groups of up to five until seven weeks of age when they were separated into pairs. One male was introduced to each cage, and each morning the dams were checked for the presence of a copulatory plug. Those dams that had a copulatory plug were removed from the cage and placed into group cages. The dams that did not have a copulatory plug were left with a male until a plug was observed.

We used dexamethasone to reduce stress in these pregnant dams in late gestation. Dexamethasone is a synthetic GC that simulates an artificial low stress environment [52,53] and is used during late gestation in humans to reduce the risk of respiratory distress syndrome in premature babies [22]. Fetal effects from the simulated low stress environment are expected to be exaggerated because dexamethasone is not metabolized by the placenta [54]. Thus, there are fewer maternal GCs crossing the placenta as a result of dexamethasone interacting with the mother's body, as well as free dexamethasone entering the fetus and blocking its naturally occurring GCs. Combined, these effects result in perceived low stress levels for offspring.

At day 16 after the presence of a copulatory plug, 1.0 µg ml^−1^ of dexamethasone (as used by [52]) was added to the drinking water of 22 dams, and this was replaced with fresh water after 3 days. Although this method results in variable dosages, it eliminates any increase in GCs from the stress of handling and injections [53], which potentially could negate the treatment [52]. Water-soluble dexamethasone is provided in a complex with 2-hydroxypropyl-β-cyclodextrin. Therefore, we had 10 dams whose water was treated with 14.4 µg ml^−1^ 2-hydroxypropyl-β-cyclodextrin as a vehicle control, to equally match the amount of vehicle that was required to deliver 1.0 µg ml^−1^ of dexamethasone. The water of eight dams was left untreated, as the negative control.

As close as possible to birth and at least within 10 h, the pups were counted to record litter size in case of infanticide. These pups are considered to be the focal animals; the sperm sex ratios and offspring sex ratios produced by them are a means of determining the influence that maternal stress had. At 21 days after birth, the focal pups were sexed via visual examination of the anogenital distance and separated into single sex group cages. To avoid pseudo-replication, only one focal female and one focal male from each litter were kept as the focal animals. At seven weeks of age, the focal pups were considered adult, and body measurements (table 1) were taken.

**Table 1.** Variables measured from BALB/c mice, used in a mating trial to determine whether maternal effects (*in utero* treatment with dexamethasone) have the ability to constrain sex allocation in laboratory mice. Physical body measurements were taken at maturity (seven weeks of age).

variable	description
body condition	calculated from the residuals of an ordinary least-squares linear regression of body mass and pes length [55]. Pes length is measured using digital callipers
anogenital distance	calculated as the distance between the anus and the genital opening. Measuring using digital callipers
digit ratio	digit ratio was calculated as the ratio of second to fourth digit on the hind right foot. Digit length is measured using digital callipers from the tip of the toe to the base of the footpad. Observers were blind to the treatment of the animal
blood glucose	blood glucose was measured using an Accu-Chek Performa Nano glucometer, from blood collected via tail tipping

### 2.2. Breeding of focal females

Focal females were housed in pairs with an unrelated male until a copulatory plug was noted, after which females were weighed and blood glucose tested. Three days later the blood glucose test was repeated, to calculate the change in peri-conceptual blood glucose level. Focal females were allowed to give birth naturally and pups were again sexed using anogenital distance. Seven focal females did not conceive, and a further two committed infanticide prior to offspring sexing and were removed from the analysis. The final sample size was 31 (figure 1). The sex ratio of the resultant litter was recorded.

### 2.3. Sperm collection from focal males

Focal males were sacrificed via cervical dislocation at between 67 and 74 days of age. The left epididymis and vas deferens were dissected into 0.5 ml cryopreservation media (18% raffinose + 3% skim milk). The semen was squeezed from the vas deferens using tweezers and allowed to swim out of the epididymis through lateral incisions. The resultant sperm suspensions were stored in straws and cryopreserved in liquid nitrogen.

### 2.4. Fluorescence *in situ* hybridization on sperm

The full methods are described in Edwards *et al*. [56]. Briefly, the sperm samples were washed and fixed to glass slides, decondensed and treated with pepsin prior to denaturation in 70% formamide. The X-chromosome probes were labelled with Cy3 and Y-chromosome probes with biotin. Denatured probes were added to the slides and hybridizations were performed in a warm, moist chamber for 24–48 h. Slides were washed and detection of the Y-chromosome probe was performed using avidin-fluorescein isothiocyanate (FITC), prior to counterstaining the sperm heads with 4′6-diamidion-2-phenylindole ml^−1^ (DAPI) and mounting using an anti-fade solution (Vectashield, Vecta Laboratories, CA). Sperm were observed using a Leica DMRXA fluorescence microscope, with Cy3, FITC and DAPI specific filters. A minimum of 500 spermatozoa were counted per individual, from images collected using Leica QFISH with a cooled CCD camera through ×40 or ×63 oil-immersion objectives.

Of the 40 initial litters, four did not produce any males, three sperm samples were destroyed during transportation, and one sample failed to hybridize sufficiently for analysis, resulting in 33 focal males (figure 1).

### 2.5. Statistics

All analyses were performed in R v. 3.2.2 [57].

### 2.6. Focal female offspring sex ratio analysis

Binomial generalized linear models with an intercept of 1 were run to determine whether the treatment group or either control group presented with sex ratios different to the predicted 50 : 50 ratios. These results are presented as 95% CIs on the estimate.

A generalized linear model with binomial error was run to determine whether peri-conceptual change in glucose, treatment or body condition influenced the sex ratio of offspring. This model also included an interaction effect between peri-conceptual glucose and treatment. While a multivariate analysis of variance (MANOVA) was run to determine whether the treatment had any effect on the physical body measurement of focal animals. An analysis of variance (ANOVA) was also run to determine whether litter size varied with treatment.

### 2.7. Focal male sperm sex ratio analysis

A full generalized linear model with binomial error was run to determine whether treatment or body condition influences the sex ratio of sperm. While a MANOVA was run to determine whether the treatment had any effect on the physical body measurement of focal animals.

## 3. Results

### 3.1. Litter sex ratios

**Figure 2.**
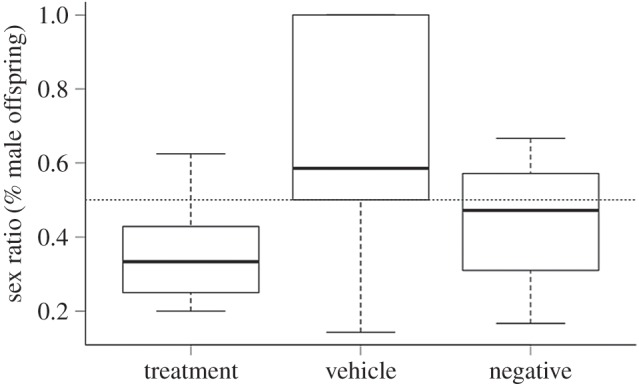
Female mice that receive dexamethasone treatment *in utero* produce litters with sex ratios that are lower than the expected 50 : 50 ratio (GLM: −0.943, −0.161), but females who received the vehicle or untreated water did not (GLM vehicle control: −0.922, 0.738; GLM negative control: −0.798, 0.274). The dotted line indicates the expected 50 : 50 ratio.

The treatment group produced sex ratios that were significantly lower than the predicted 50 : 50 ratio (generalized linear model (GLM): −0.943, −0.161; figure 2), whereas neither control group differed from parity (GLM negative control: −0.798, 0.274; GLM vehicle control: −0.922, 0.738).

**Figure 3.**
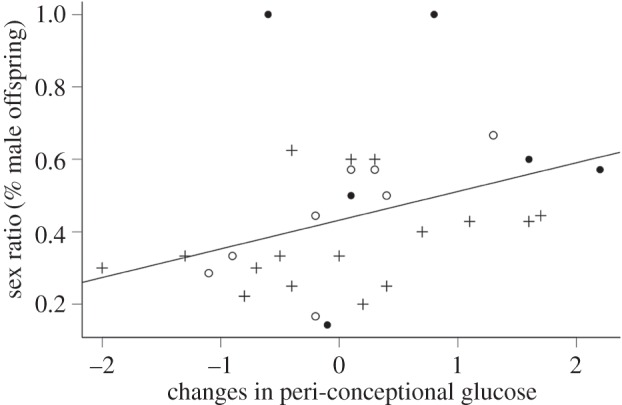
The linear relationship between sex ratio (as percentage of male offspring) and peri-conceptional blood glucose changes from day 0 to day 3 after confirmed copulation in laboratory mice (GLM, Pr (>*χ*) = 0.03). Crosses represent the sex ratios of females who received dexamethasone treatment during late development (*in utero*). Filled circles represent females who received the vehicle control and open circles represent females that did not receive any treatments.

The sex ratio of offspring was significantly influenced by peri-conceptual change in glucose (Pr (>*χ*)_1,29_ = 0.033; figure 3), but not by treatment (Pr (>*χ*)_2,27_ = 0.676) or body condition (Pr (>*χ*)_1,26_ = 0.915). There was also no interaction effect between the change in peri-conceptual glucose and treatment (Pr (>*χ*)_2,24 _= 0.554). The treatment did not result in a change in litter size (*F*_2,28_ = 3.174, *p* = 0.057); however, there was a slight trend for the vehicle control group to have smaller litters. The treatment also did not influence the physical and physiological body measurements of the focal animals (*F*_10,48_ = 0.955, *p* = 0.493).

### 3.2. Sperm sex ratios

The sperm sex ratio was not significantly influenced by treatment (Pr (>*χ*)_2,30_ = 0.192) or body condition (Pr (>*χ*)_1,29_ = 0.488). There was also no effect of treatment on any physical or physiological body measurement of the focal males (*F*_8,56 _= 0.975, *p* = 0.477).

## 4. Discussion

Maternal effects altered focal female sex ratios, but not the X- and Y-chromosome ratio in focal male sperm. Females that received the dexamethasone treatment during late-gestational development gave birth to litters with sex ratios lower than the predicted 50 : 50 ratio, with no change to litter size. However, increases in blood glucose were more strongly associated with an increase in male offspring than treatment *per se*, suggesting that environmental interactions with glucose metabolism may be more influential than maternal effects.

The developmental impacts of late-gestational maternal stress manipulation influence stress responses and glucose metabolism in later life [22]. Embryonic female guinea pigs exposed to dexamethasone *in utero* have increases in glucocorticoid receptor and mineralocorticoid receptor mRNA in all regions of their hippocampus and altered GC levels, which are lower in the luteal phase but higher during oestrous [22]. However, increases in cortisol are associated with hepatic gluconeogenesis [42] and an overall increase in glucose [58]. Therefore, the lowering of cortisol levels during the luteal phase and the observed increase in female offspring might be better explained through the glucose hypothesis [5], through associated low levels of gluconeogenesis, and therefore, an overall decrease in free glucose.

In this study, the focal females that had an increase in blood glucose levels over the time of conception and early gestation give birth to more sons. This provides further evidence in support of the glucose hypothesis [5], where early blastocyst females survive better in low glucose environments, and males in high glucose environments [45]. Change in blood glucose levels significantly influence sex ratios while treatment only did so indirectly through an interaction with glucose levels, probably due to the delivery method, because drinking water results in variable dosages [52]. However, as dexamethasone was used to simulate low stress, variable dosage was preferable to negating the treatment from injection-induced stress [52,53].

The possibility of maternal effects constraining a father's sperm production has not been previously investigated. No significant shift in sperm sex ratios of the focal males is unsurprising, as we do not anticipate that stress or changes to HPA axis functioning should affect sperm production. Unlike mothers, mammalian fathers do not require large energetic investment in the production of gametes [59], or even in the offspring themselves [59], and therefore, changes to stress pathways are unlikely to influence paternal sex allocation. However, research into paternal sex allocation and the possibility of adaptive control by fathers is limited ([9], but see [60–62]), and it is unknown under what circumstances paternal sex allocation could occur [9,56], although James [63] has suggested a role for pre-mating androgens in fathers.

There were no changes to the physical appearance of either sex offspring, even though previous studies on gestational dexamethasone have shown variation in physical characteristics (reviewed in [64]). Many of the studies that have presented offspring with physical changes have used much larger intravenous or subcutaneous dosages, and even multiple dosages, which leads to greatly exaggerated effects [64]. In comparison, our dosage was high enough to have physiological effects on subsequent sex ratios (suggesting changes to underlying physiology) but not enough to have deleterious effects on offspring morphological development. In addition, we found no evidence that testosterone was linked to sex allocation. We measured both the digit ratio and the anogenital distance of the mice, which are indicative of the female's prenatal androgen exposure [65], but neither of these were correlated with sex ratio. There is contention regarding the use of digit ratios as androgen exposure indicators [66], and, therefore, although our data show no support for a role of testosterone, we cannot rule out a role for testosterone influencing sex ratios.

We have shown that the gestational environment results in female offspring whose physiology is altered in a way that affects her reproductive functioning as an adult, which could influence the success of management and captive breeding programmes. Changes to female physiological pathways due to maternal effects can constrain maternal sex allocation in subsequent generations, producing females that respond differently to the same environmental conditions, despite appearing otherwise similar.

## Data accessibility

Data available from the Dryad Digital Repository: http://dx.doi.org/10.5061/dryad.b18gv.
